# Floral *KNOX* Expression Supports Parallel Evolution of Nectar Spurs in Snapdragon Relatives (Antirrhineae: Plantaginaceae)

**DOI:** 10.1111/ede.70061

**Published:** 2026-09-14

**Authors:** Steven Dodsworth, Grace Heath, Jack Smith, Jelesha Gray, Thomas Davies, Glaucia Proszkowiec, Omoyemwen Emokpae, Denise Patel, Thomas Holt, Bethany Hörnfeldt, Maarten Christenhusz

**Affiliations:** ^1^ Institute of Structural and Molecular Biology, School of Natural Sciences, Birkbeck University of London London UK; ^2^ School of the Environment and Life Sciences University of Portsmouth Portsmouth UK; ^3^ School of Life Sciences University of Bedfordshire Luton UK; ^4^ Royal Botanic Gardens, Kew Richmond UK; ^5^ Hortus Botanicus Technical University Delft Delft The Netherlands

**Keywords:** *KNOX*, nectar spur, petal spur, plant‐pollinator interactions, transcription factors

## Abstract

Nectar spurs are tubular outgrowths of perianth tissue—a key innovation that has evolved many times independently in angiosperms. These novel floral structures are tightly linked to pollination and pollinator shifts may explain their role in increasing diversification rates. In the tribe Antirrhineae (Plantaginaceae), nectar spurs on the ventral petal have convergently evolved four times, in the *Kickxia* clade, *Cymbalaria*, *Chaenorhinum*, and *Linaria*. Here we explore the molecular mechanism underpinning this convergence, by investigating two Class I *KNOX* genes (*HIRZ* and *INA*) across Antirrhineae. Such *KNOX* genes are canonically expressed in the shoot apical meristem (SAM). *HIRZ* and *INA* were originally identified in mutants of *Antirrhinum majus* (snapdragons) whereby ectopic floral expression led to mutated spur‐bearing petals; subsequently *HIRZ/INA* were shown to have floral expression in the naturally spur‐bearing relative *Linaria vulgaris* (toadflax). Orthologues of *HIRZ* and *INA* were isolated here from *Kickxia elatine*, *Cymbalaria muralis*, and *Chaenorhinum origanifolium*, and confirmed through phylogenetic and gene structure analyses. Similar expression patterns were found in all three species, with moderate‐high expression of both *HIRZ* and *INA* in floral buds and mature flowers, consistent high expression in apices (i.e., the SAM), and low expression in leaves. Higher levels of expression were found in floral buds compared to leaves (on average 75‐86‐fold higher) for *INA* and *HIRZ*, respectively. Together these data strongly suggest that the four independent evolutions of the petal spur in Antirrhineae are a result of parallel evolution, through the independent recruitment of similar underlying molecular mechanisms.

## Research Highlights

- Nectar spurs have evolved four times across the Antirrhineae.
- Spur development on ventral petals has been associated with floral *KNOX* gene expression.
- *KNOX* expression across evolutionary events suggests parallel molecular evolution of spurs.

## Graphical abstract

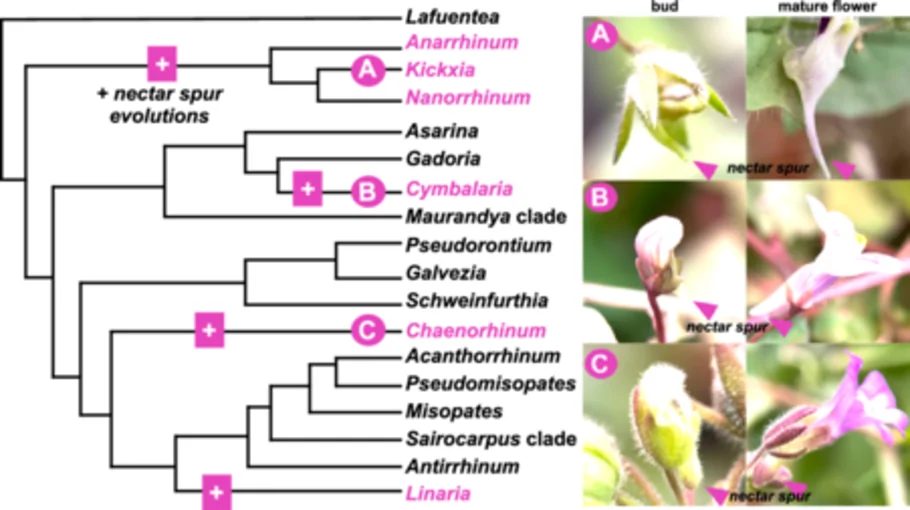

## 1 Introduction

Angiosperms are defined by several key innovations, most notably the presence of flowers. Floral morphology is linked to reproductive success through plant‐pollinator interactions. Traits affecting pollination are associated with reproductive isolation and thereby higher speciation and diversification rates in many lineages, although this is complex and variable (Van der Niet and Johnson 2012; Van der Niet et al. 2014; Benton et al. 2021). Nectar spurs are one such floral trait, tube‐like outgrowths of perianth tissue, often petal or sepal tissue, that store nectar and provide this reward to pollinators. Nectar spur length variability has been tightly linked to pollination syndrome, and the presence of nectar spurs as well as length differences of spurs have both been implicitly and explicitly tied to increased diversification rates (Hodges and Arnold 1995; Whittall and Hodges 2007; Fernández‐Mazuecos et al. 2019; Li et al. 2024). However, spurred plants may be visited by both short‐ and long‐proboscid visitors and therefore may sometimes function in a more complex manner ecologically than typically expected (Vlašánková et al. 2017). Nectar spurs have evolved many times across the angiosperms, and are found in 12 orders and 23 families of both monocot and eudicot groups, including Orchidaceae, Papaveraceae, Ranunculaceae, Balsaminaceae, Violaceae, and Plantaginaceae (Yang and Jin 2023; Li et al. 2024). This repeated convergent evolution of nectar spurs suggests the presence of strong selection pressures/selective advantage. Despite this morphological convergence, not many taxa have been studied in terms of detailed spur development nor underlying developmental genetic programmes.

Fundamentally the creation of a nectar spur on a flower requires a new plane of cell division and expansion (Wessinger and Hileman 2020), to create a three‐dimensional structure on a novel axis (relative to the orientation of petal or sepal tissue). There are many factors that influence cell division and organ specification in plants, and this may explain the possibility of multiple molecular players and distinct developmental programmes that are able to produce spur structures in different lineages. In both *Linaria* and *Aquilegia* the development of the spur is governed by a combination of both cell division and cell expansion, although the relative contribution of each process and its timing vary. Spur length variation in *Linaria* was found to result from differences in cell division (i.e., cell number) rather than cell expansion, with 3x more cells in a longer‐spurred taxon and only 1.3x larger cells (Cullen et al. 2018). In contrast, spur lengths in *Aquilegia* are mostly governed by anisotropic cell expansion over time, which accounts for almost all variation in spur length across the genus (Puzey et al. 2012).

**Figure 1:**
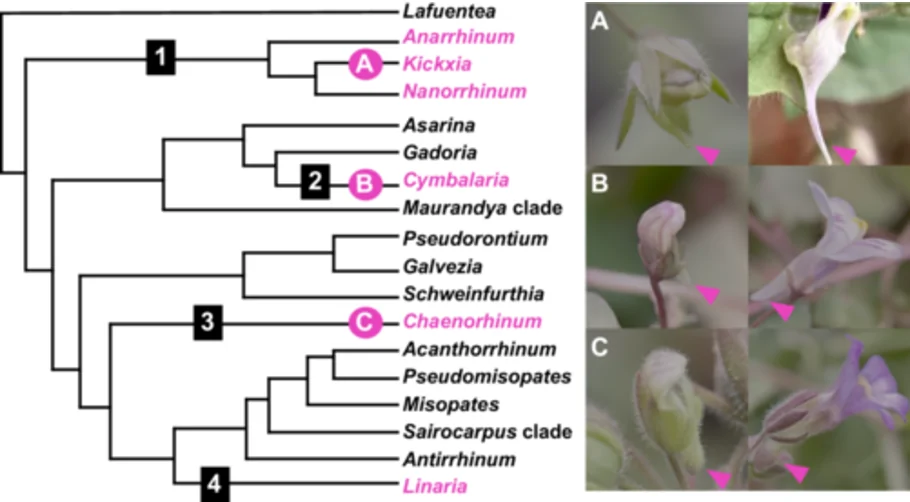
Summary phylogenetic tree showing four independent evolutions of nectar spurs in Antirrhineae (adapted from Fernández‐Mazuecos et al. 2019), with spur‐bearing genera highlighted in pink. Photographs show floral buds and mature flowers of the three taxa studied here. A, *Kickxia elatine*; B, *Cymbalaria muralis*; C, *Chaenorhinum origanifolium*. Nectar spurs are indicated by pink arrowheads. [Color figure can be viewed at wileyonlinelibrary.com]

In Lamiales there are at least five families with nectar spurs (Lamiaceae, Lentibulariaceae, Plantaginaceae, Pedaliaceae, Scrophulariaceae), in each case extensions of corolla, that is, petal, tissue (Yang and Jin 2023). This suggests the potential for parallel evolution, which may naturally lead to several gains/losses of spur morphology over time. Within Plantaginaceae, the tribe Antirrhineae includes approximately 3–400 species, of which approximately 80% have nectar spurs (Fernández‐Mazuecos et al. 2019). Morphologically these are all very similar as outgrowths of the ventral petal tissue. Within the Antirrhineae, nectar spurs have evolved four times independently over the past ~5–20 Mya, in the *Kickxia* clade, *Cymbalaria*, *Chaenorhinum*, and *Linaria* (Figure 1). Spurred lineages were loosely associated with increased diversification rates relative to unspurred lineages, and in *Linaria*, the largest clade of spurred Antirrhineae, there was a convincing relationship between spur length changes and speciation (Fernández‐Mazuecos et al. 2019). A clear question that follows is whether or not these independent evolutions of spurs are underpinned by the same/similar genetic mechanisms (i.e., parallelism).

Class I KNOX transcription factors were previously shown to have a role in nectar spur development in Antirrhineae. In *Antirrhinum majus* two mutants were described with dominant gain‐of‐function mutations in *KNOX* genes—*Hirzina‐*d153 (in *AmHIRZ*) and *Invaginata*‐d1 (in *AmINA*). These resulted in ectopic expression of *AmHIRZ*/*AmINA* that caused floral phenotypes with outgrowths on the ventral petal resembling nectar spurs found in other Antirrhineae (Golz et al. 2002). In *Linaria vulgaris*, which naturally bears long (6–13 mm) nectar spurs on its ventral petal, expression of *LvHIRZ* and *LvINA* was found natively in floral tissues (Box et al. 2011). *HIRZ* and *INA* both encode class I KNOX transcription factors (TFs), potentially the product of an historical gene/genome duplication event in Plantaginaceae. KNOX TFs have canonical roles in maintenance of cell division within meristematic tissues (Dodsworth 2009; Maksimova et al. 2021) and in the formation of leaflets/lobes during compound leaf development (Wilson‐Sánchez et al. 2022). In floral tissue it is presumed they may provide a similar role by stimulating cell division for the novel axis of spur growth (Box et al. 2011; Box et al. 2012).

Here we investigate the expression of *HIRZ* and *INA* orthologues in three independent evolutions of spurs in tribe Antirrhineae, distinct from *Linaria*. We isolated and confirmed orthologous *HIRZ* and *INA* gene sequences from *Kickxia*, *Cymbalaria* and *Chaenorhinum* and used a (q)RT‐PCR approach to investigate gene expression. We specifically ask: (i) Are *HIRZ/INA* orthologues expressed in floral buds and mature flowers?; (ii) Is *HIRZ/INA* expression higher in floral tissues than leaves? and (iii) Are expression patterns similar across taxa and tissues (apex, leaf, floral buds, mature flowers)?

## 2 Materials and Methods

### 2.1 Plant Materials and Nucleic Acid Isolation

*Chaenorhinum origanifolium* (L.) Kostel. ‘Blue Dream’ was purchased from Farmyard Nurseries (Llandysul, UK). *Cymbalaria muralis* G.Gaertn., B.Mey. & Scherb. was collected from wild populations in Portsmouth (50.7872, −1.0940), and *Kickxia elatine* (L.) Dumort. from a population at Wakehurst Place, West Sussex (51.0686, −0.0896). Voucher specimens for all taxa are held at BBK. Nucleic acids were isolated using the LogSpin method (Yaffe et al. 2012) and EZ‐10 RNA spin columns (NBS Biologicals Ltd., Huntingdon, UK) for RNA, and Qiagen DNeasy plant kit for genomic DNA (Qiagen, Manchester, UK), from approximately 100 mg of tissue per extraction. Tissues were pooled for RNA extraction. Floral buds were harvested at a stage when the spur was clearly visible pre‐opening (Figure 1); mature flowers were harvested at anthesis; apices were harvested from young growing tips as vegetative buds including some primordia tissue; and mature leaves that had approached mature size (relative to the rest of the plant).

### 2.2 *KNOX* orthologue isolation and confirmation

**Figure 2:**
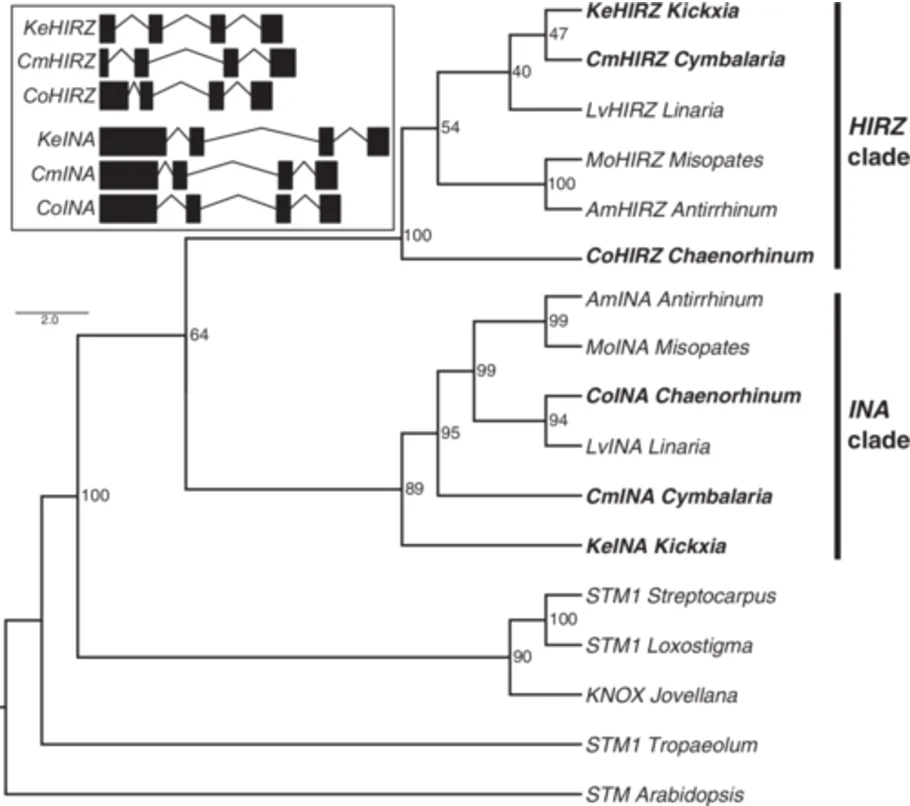
Maximum likelihood tree of *HIRZ* and *INA* orthologues from Antirrhineae, with representative *STM*‐like sequences from Lamiales and *Arabidopsis STM* as an outgroup. Sequences in bold were newly generated in this study. Support values represent 1000 bootstrap replicates. Inset shows approximate exon/intron gene structures reconstructed from genomic mapping, scaled proportionately.

Genomic sequence information for the three taxa was obtained through low‐coverage whole‐genome sequencing using Oxford Nanopore Technologies nanopore sequencing. Briefly, 1 μg of genomic DNA was used as input for ligation library preparation (SQK‐NBD‐114 kits). Reads were basecalled using the dna_r10.4.1_e8.2_400bps_sup@v5.2.0 model in Dorado basecaller post‐sequencing, and demultiplexed. Reads were then mapped to *LvHIRZ* and *LvINA* using minimap2 (Li 2018) and map‐ont settings, with an average coverage of 8x. Finally, mapped alignments were investigated manually, and genomic sequences for paralogues were assembled in Geneious Prime 2023.2.1 (https://www.geneious.com). Long sections of coding sequences (partial CDS, exon 1 through 4) were subsequently isolated through PCR amplification of cDNA. Gene structure was assessed by comparison to *Arabidopsis STM* at the protein level, due to well‐annotated genomic and transcript sequences, and by subsequent comparison of transcript and genome sequences. Gene structure diagrams were plotted using the package genemodel in R version 4.1.2 (R Core Team, 2021). The nucleotide coding sequences of *HIRZ* and *INA* orthologues were aligned with *Linaria*, *Antirrhinum*, and outgroup sequences (Supporting Information Table S1) using MAFFT with E‐INS‐i settings (Katoh et al. 2019). Alignments were trimmed using Gblocks with default settings (Talavera and Castresana 2007). A maximum likelihood tree was computed using RAxML (Stamatakis 2014) with GTR model and 1,000 bootstrap replicates. Orthology was confirmed based on the recovery of the two distinct (*HIRZ* and *INA*) clades with high support (Figure 2).

### 2.3 Expression Analyses

Total RNA extracts were DNase I‐treated (Thermo Fisher Scientific, Basingstoke, UK) in order to remove genomic DNA, and further tested for any residual trace DNA using an nrITS PCR on the RNA extraction, prior to first strand synthesis. Synthesis of cDNA was performed using UltraScript 2.0 (PCR Biosystems Ltd., London, UK) with oligo‐d(T) primers and cDNA was diluted 1:5 in sterile water, prior to PCR. Primers for RT‐PCR were designed to span an intron, in order to confirm cDNA‐specific (i.e., shorter) amplification products. A full list of primer sequences used is given in Supporting Information Table S2. *Tubulin* alpha5‐chain was used as a housekeeping gene (*TUA5*), initially using primers designed for *Linaria* (Box et al. 2011) and subsequently redesigned specifically for each taxon. RT‐PCR and qRT‐PCR were performed using 3 μl and 1 μl of 1:5 diluted cDNA template, respectively. RT‐PCR was carried out over 35 cycles and qRT‐PCR over 40 cycles. Products were visualized on 2% agarose gels. Selected RT‐ and qRT‐PCR products from all taxa were Sanger sequenced to check specificity. We subsequently performed qRT‐PCR in order to quantify the relative expression of *HIRZ* and *INA*, using qPCRBIO SyGreen Blue Mix with Lo‐ROX (PCR Biosystems Ltd., London, UK) and performed on a Bio‐Rad CFX Duet system (Bio‐Rad Laboratories Ltd., Hertfordshire, UK). Each qPCR run included all genes for that set of tissues, in order to minimize any assay‐specific amplification bias and individual run changes to cycle threshold (C_T_). Quantitative expression was calculated as ΔC_T_ values, whereby ΔC_T_ = C_T_ (Housekeeping reference gene—*TUA5*) – C_T_ (Gene of interest—*HIRZ* or *INA*). This is such that larger ΔC_T_ values naturally represent greater expression of the gene of interest. In each case three biological replicates are shown, which in turn are the means of three technical replicates. We visualize relative expression as the raw Δ C_T_ values, as these are approximately normally distributed and on a log scale, thereby showing both up‐ and down‐regulation equally. Exponentiating the ΔC_T_ values (i.e., 2^ΔCt^), is not favored as this includes an unknown, assay‐specific constant and is therefore not directly the log of relative expression. Data were visualized in R using the package ggplot2 (Wickham 2016). Data were normality checked using Shapiro‐Wilk tests, and t‐tests were performed on the pooled ΔC_T_ values, for both *HIRZ* and *INA*, for floral tissues relative to leaf in each case, with an alpha threshold of *p* < 0.05. Fold‐changes are only quoted in the text as comparisons between floral tissues and leaf tissue (i.e., exponentiates of ΔΔC_T_(ΔC_T_[floral tissue]‐ ΔC_T_[leaf])).

## 3 Results and Discussion

### 3.1 Orthologous *KNOX* Genes From Across Antirrhineae

We successfully isolated both *HIRZ* and *INA* orthologues from *Kickxia* (*KeHIRZ*, *KeINA*), *Cymbalaria* (*CmHIRZ*, *CmINA*), and *Chaenorhinum* (*CoHIRZ*, *CoINA*; Figure 2). A four‐exon structure was found across all taxa, similar to *Antirrhinum* and *Linaria* (Golz et al. 2002; Box et al. 2011). *HIRZ* orthologues (*KeHIRZ*, *CmHIRZ*, *CoHIRZ*) all contained four exons of comparable size across taxa (Figure 2; Supporting Information Figure S1), except that *Cymbalaria* and *Kickxia* showed a reduced size of the first exon. *HIRZ* genomic sequences (exon 1‐exon 4) ranged from 3180 to 3621 bp. Similarly *INA* orthologues (*KeINA*, *CmINA*, *CoINA*) all contained four exons of very similar sizes across all taxa (Figure 2). However, intron size was significantly more variable, with *Kickxia* showing an expanded second intron (Figure 2). *INA* genomic sequences ranged from 4342 to 5270 bp. Protein sequences and domains are shown in Supporting Information Figure S1, confirming gene function.

Both *HIRZ* and *INA* were additionally found in a recent chromosome‐level genome assembly of *Misopates orontium* (non‐spur bearing, Antirrhineae, Figure 2; Christenhusz et al. 2024) but were not apparent in *Penstemon* (tribe Cheloneae) or *Veronica* (tribe Veroniceae). This suggests that the gene duplication that led to *HIRZ* and *INA* paralogues may have occurred early in the evolution of the tribe Antirrhineae (c. 50 Mya), but likely not earlier in the entire Plantaginaceae family.

**Figure 3:**
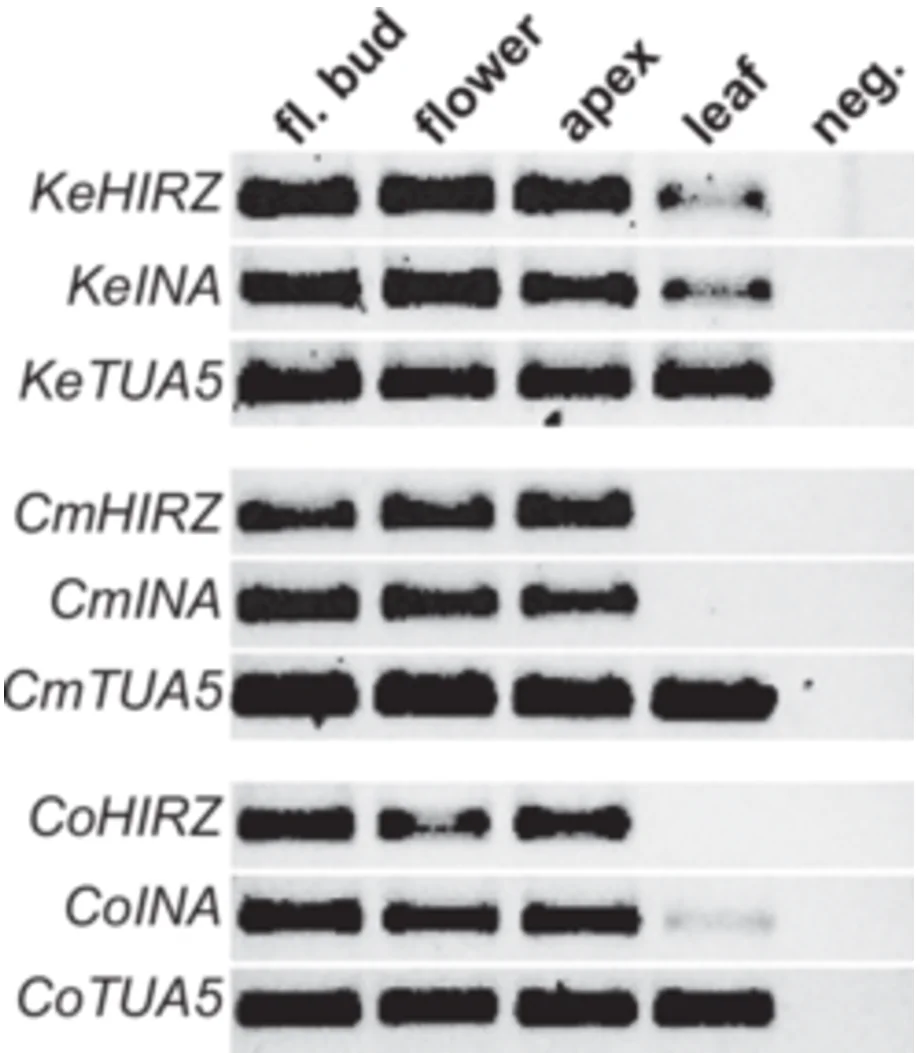
Gene‐specific RT‐PCR of *HIRZ* and *INA* orthologues relative to *TUA5* in floral and vegetative tissues. *Ke, Kickxia elatine*; *Cm*, *Cymbalaria muralis*; *Co*, *Chaenorhinum origanifolium*.

**Figure 4:**
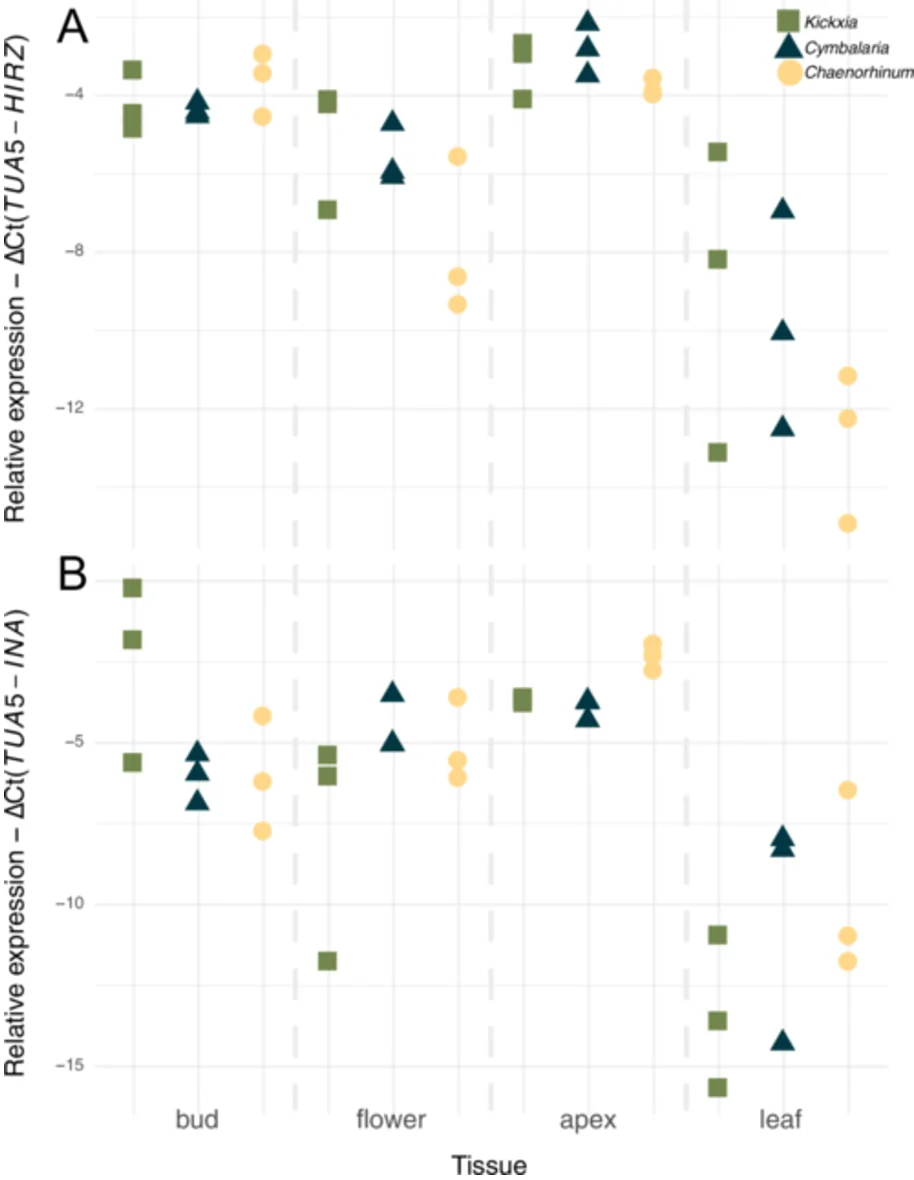
Quantitative expression of A—*HIRZ* and B—*INA* orthologues relative to *TUA5* in floral and vegetative tissues. Relative expression is shown as ΔCt(*TUA5*‐*GOI*), such that higher values intuitively represent greater expression, on a log scale. Individual biological replicates are shown for each tissue and taxon per gene. [Color figure can be viewed at wileyonlinelibrary.com]

High levels of expression of both *HIRZ* and *INA* were found in apices (Figure 3), in most cases higher than or equal to that in floral tissues (Figure 4). Given the strong expression of both genes in the apices of all taxa investigated it is likely that the ancestral role of these Class I *KNOX* genes is in maintenance of the stem cell population in apical meristems, as found in many other eudicot taxa and also in *A. majus* (Golz et al. 2002; Dodsworth 2009; Maksimova et al. 2021). Here they act to help maintain cell division. This notably contrasts with previous results in *Linaria* (Box et al. 2011), which showed relatively low‐moderate expression in apices. Overlapping expression patterns in all taxa also likely indicate some redundancy in *HIRZ/INA* function.

### 3.2 *KNOX* Expression Patterns in Antirrhineae Flowers

Here we show strong expression of *HIRZ* and *INA* orthologues in floral tissues (Figure 3) for three other taxa from Antirrhineae—*Kickxia*, *Cymbalaria* and *Chaenorhinum*—that collectively represent three independent evolutions of spurs (Fernández‐Mazuecos et al. 2019) relative to *Linaria* (Box et al. 2011). Expression was generally higher in floral buds than mature flowers, which showed greater variability, and expression was very low to absent from mature leaves (Figure 3, Figure 4). Expression of *HIRZ* orthologues was significantly higher in floral buds compared to leaves (Figure 4), on average 86‐fold higher (ΔΔC_T_ of 6.43; *p* = 0.0002) and 20‐fold higher for mature flowers compared to leaves (ΔΔC_T_ of 4.34; *p* = 0.003). Expression of *INA* orthologues was also significantly higher in floral buds compared to leaves, on average 75‐fold higher (ΔΔC_T_ of 6.23; *p* = 0.0003) and 40‐fold higher for mature flowers compared to leaves (ΔΔC_T_ of 5.33; *p* = 0.001). This is consistent with high floral expression of *LvHIRZ* and *LvINA* found previously for *Linaria* (Box et al. 2011), although Box et al. found generally lower levels of *LvINA* expression compared to *LvHIRZ*, whereas we found comparable levels of expression in all taxa studied here (Figure 4).

Whether or not floral expression of *HIRZ* and *INA* may itself be due to the same proximate mechanism, presumably *cis* changes in upstream regulatory regions (cf. original mutants in *Antirrhinum*) requires further investigation. This will require a larger number of high‐quality genomes sampled across the Antirrhineae, in order to see whether insertions in *cis* regulatory regions may be a common mechanism underpinning expression changes in *KNOX*. It would also be interesting to investigate whether transposable elements play a natural role in this process.

It has been shown previously that ectopic expression of *AmHIRZ* in floral tissues led to the development of a novel floral tube resembling a small spur, and ectopic expression of *AmINA* led to a duplicated corolla tube (Golz et al. 2002). Wild‐type *Antirrhinum*, however, only harbors a gibba, a small pouch on the ventral petal. Similarly, the heterologous expression of *LvHIRZ* and *LvINA* in tobacco (*Nicotiana tabacum*) led to ectopic spur‐like growths on the corolla tube (Box et al. 2011). It remains to be tested whether silencing of *HIRZ/INA* would lead to the loss of spurs in a naturally spur‐bearing Antirrhineae taxon, but this would make a logical next step to functionally test the importance of HIRZ and INA proteins in spur development.

### 3.3 *KNOX* Expression Suggests Parallel Molecular Evolution of Nectar Spurs

The similar pattern of expression of *HIRZ* and *INA* across four independent evolutions of the spur suggests that a common molecular mechanism underpins spur development in Antirrhineae. KNOX transcription factors are typically not expressed in floral tissues, but have established roles in maintenance of the stem cell population in the shoot apical meristem (Maksimova et al. 2021), and also in defining the boundaries of lobed and compound leaves (Wilson‐Sánchez et al. 2022). Their role outside of the SAM in organ primordia and differentiation appears to be in regulating cell division in order to create differences in the patterning of organ boundaries. Late reactivation of *KNOX* is also hypothesized to have a role in other outgrowths of petals such as coronas (Appleton and Kramer 2024). Rather intriguingly *KNOX* seemingly have little to no role in spur development of other well‐studied taxa that possess nectar spurs, such as *Aquilegia* (Yant et al. 2015), in which C2H2 zinc‐finger TFs have been shown to play a significant causative role in development (Ballerini et al. 2020). Likewise in *Tropaeolum KNOX* play a role in cell indeterminancy but not specifically in spur development (Martínez‐Salazar et al. 2023.) Yet the number of angiosperm taxa that have nectar spurs is approximately 3400 (Li et al. 2024) and most of these have not been studied in any detail.

A fundamental question in evolutionary biology is whether repeated evolution is the result of similar underlying genetic mechanisms or not, especially where evolution repeats itself over relatively short timeframes. It appears that nectar spur evolution within Antirrhineae represents another instance of this particular kind of convergent evolution, i.e., parallel evolution/parallelism (*sensu* Scotland 2011)—genetic homoplasy that underpins phenotypic homoplasy of the trait in question. Such parallel molecular evolution also hints at the potential evolvability of the trait in question, which may be underpinned by a relatively simple developmental switch.

## 4 Conclusions and Future Perspectives

*HIRZ* and *INA* expression represents a common developmental signature that strongly suggests similar molecular mechanisms may underpin the development of nectar spurs across four independent evolutionary events in Antirrhineae. However, whilst these KNOX TFs may be a necessary component they are likely not sufficient and functional analyses are required to decipher their precise role. A comparative transcriptomic approach could further untangle the regulatory network underpinning spur development (e.g., an extension of Cullen et al. 2023), comparing spurred and non‐spurred close relatives. It would also be worthwhile investigating other genes identified as playing a potentially significant role, such as *CCD33* (Cullen et al. 2023) and *POP* (Ballerini et al. 2020). Furthermore, a single‐cell based approach could provide more nuanced fine‐scale expression patterns in particular regions of the ventral petals. This may give additional evidence on the fundamental molecular mechanism governing spur development.

## Supporting information


**Figure S1:** Protein alignment of HIRZ and INA orthologues, with representative STM‐like sequences.

**Table S1:** Gene sequences used for phylogeny.
**Table S2:** Primer sequences.

## Data Availability

These sequence data have been submitted to the European Nucleotide Archive (ENA) at EMBL‐EBI under project accession number PRJEB110527 and sequence accession numbers OZ544814‐OZ544819. The data that support the findings of this study are openly available in ENA at https://www.ebi.ac.uk/, reference number PRJEB110527.

## References

[ede70061-bib-0001] Appleton, A. D. , and E. M. Kramer . 2024. “Genetic Architecture of Novel Floral Organs.” International Journal of Plant Sciences 185, no. 3: 211–217: number. 10.1086/729359.

[ede70061-bib-0002] Ballerini, E. S. , Y. Min , M. B. Edwards , E. M. Kramer , and S. A. Hodges . 2020. “POPOVICH, Encoding a C2H2 Zinc‐Finger Transcription Factor, Plays a Central Role in the Development of a Key Innovation, Floral Nectar Spurs, Inaquilegia.” Proceedings of the National Academy of Sciences 117: 22552–22560. 10.1073/pnas.2006912117. PMC7486772 32848061

[ede70061-bib-0003] Benton, M. J. , P. Wilf , and H. Sauquet . 2021. “The Angiosperm Terrestrial Revolution and the Origins of Modern Biodiversity.” New Phytologist 233: 2017–2035. 10.1111/nph.17822. 34699613

[ede70061-bib-0004] Box, M. S. , S. Dodsworth , P. J. Rudall , R. M. Bateman , and B. J. Glover . 2011. “Characterization of *Linaria KNOX* Genes Suggests a Role in Petal‐Spur Development.” Plant Journal 68: 703–714. 10.1111/j.1365-313X.2011.04721.x. 21790812

[ede70061-bib-0005] Box, M. S. , S. Dodsworth , P. J. Rudall , R. M. Bateman , and B. J. Glover . 2012. “Flower‐Specific *KNOX* Phenotype in the Orchid *Dactylorhiza fuchsii* .” Journal of Experimental Botany 63: 4811–4819. 10.1093/jxb/ers152. 22771852 PMC3428008

[ede70061-bib-0006] Christenhusz, M. J. M. , M. F. Fay , Royal Botanic Gardens Kew Genome Acquisition Lab , et al. 2024. “The Genome Sequence of Weasel's Snout, Misopates Orontium (L.) Raf. (Plantaginaceae).” Wellcome Open Research 9: 123. 10.12688/wellcomeopenres.20995.1.

[ede70061-bib-0007] Cullen, E. , M. Fernández‐Mazuecos , and B. J. Glover . 2018. “Evolution of Nectar Spur Length in a Clade of *Linaria* Reflects Changes in Cell Division Rather Than in Cell Expansion.” Annals of Botany 122: 801–809. 10.1093/aob/mcx213. 29370374 PMC6215036

[ede70061-bib-0008] Cullen, E. , Q. Wang , and B. J. Glover . 2023. “How do You Build a Nectar Spur? A Transcriptomic Comparison of Nectar Spur Development in *Linaria vulgaris* and Gibba Development in *Antirrhinum majus* .” Frontiers in Plant Science 14: 1190373. 10.3389/fpls.2023.1190373. 37426957 PMC10328749

[ede70061-bib-0009] Dodsworth, S. 2009. “A Diverse and Intricate Signalling Network Regulates Stem Cell Fate in the Shoot Apical Meristem.” Developmental Biology 336: 1–9. 10.1016/j.ydbio.2009.09.031. 19782675

[ede70061-bib-0010] Fernández‐Mazuecos, M. , J. L. Blanco‐Pastor , A. Juan , et al. 2019. “Macroevolutionary Dynamics of Nectar Spurs, a Key Evolutionary Innovation.” New Phytologist 222: 1123–1138. 10.1111/nph.15654. 30570752

[ede70061-bib-0011] Golz, J. F. , E. J. Keck , and A. Hudson . 2002. “Spontaneous Mutations in *KNOX* Genes Give Rise to a Novel Floral Structure in *Antirrhinum* .” Current Biology 12: 515–522. 10.1016/S0960-9822(02)00721-2. 11937019

[ede70061-bib-0012] Hodges, S. A. , and M. L. Arnold . 1995. “Spurring Plant Diversification: Are Floral Nectar Spurs a Key Innovation?” Proceedings of the Royal Society of London. Series B: Biological Sciences 262: 343–348. 10.1098/rspb.1995.0215.

[ede70061-bib-0013] Katoh, K. , J. Rozewicki , and K. D. Yamada . 2019. “MAFFT Online Service: Multiple Sequence Alignment, Interactive Sequence Choice and Visualization.” Briefings in Bioinformatics 20: 1160–1166. 10.1093/bib/bbx108. 28968734 PMC6781576

[ede70061-bib-0014] Li, H. 2018. “Minimap2: Pairwise Alignment for Nucleotide Sequences.” Bioinformatics 34: 3094–3100. 10.1093/bioinformatics/bty191. 29750242 PMC6137996

[ede70061-bib-0015] Li, S. , J. Fan , C. Xue , H. Shan , and H. Kong . 2024. “Spur Development and Evolution: An Update.” Current Opinion in Plant Biology 81: 102573. 10.1016/j.pbi.2024.102573. 38896925

[ede70061-bib-0016] Maksimova, A. I. , L. Berke , M. G. Salgado , et al. 2021. “What can the Phylogeny of *Class I KNOX* Genes and Their Expression Patterns in Land Plants Tell us about the Evolution of Shoot Development?” Botanical Journal of the Linnean Society 195: 254–280. 10.1093/botlinnean/boaa088.

[ede70061-bib-0017] Martínez‐Salazar, S. , E. M. Kramer , F. González , and N. Pabón‐Mora . 2023. “Spatio‐Temporal Expression of Candidate Genes for Nectar Spur Development in *Tropaeolum* (Tropaeolaceae: Brassicales).” Annals of Botany 132: 1205–1218. 10.1093/aob/mcad164. 37864498 PMC10902891

[ede70061-bib-0018] Van der Niet, T. , and S. D. Johnson . 2012. “Phylogenetic Evidence for Pollinator‐Driven Diversification of Angiosperms.” Trends in Ecology and Evolution 27: 353–361. 10.1016/j.tree.2012.02.002. 22445687

[ede70061-bib-0019] Van der Niet, T. , R. Peakall , and S. D. Johnson . 2014. “Pollinator‐Driven Ecological Speciation in Plants: New Evidence and Future Perspectives.” Annals of Botany 113: 199–212. 10.1093/aob/mct290. 24418954 PMC3890394

[ede70061-bib-0020] Puzey, J. R. , S. J. Gerbode , S. A. Hodges , E. M. Kramer , and L. Mahadevan . 2012. “Evolution of Spur‐Length Diversity in *Aquilegia* Petals is Achieved Solely Through Cell‐Shape Anisotropy.” Proceedings. Biological sciences 279: 1640–1645. 10.1098/rspb.2011.1873. 22090381 PMC3282339

[ede70061-bib-0021] Scotland, R. W. 2011. “What is Parallelism?” Evolution and Development 13: 214–227. 10.1111/j.1525-142X.2011.00471.x. 21410877

[ede70061-bib-0022] Stamatakis, A. 2014. “RAxML Version 8: A Tool for Phylogenetic Analysis and Post‐Analysis of Large Phylogenies.” Bioinformatics 30: 1312–1313. 10.1093/bioinformatics/btu033. 24451623 PMC3998144

[ede70061-bib-0023] Talavera, G. , and J. Castresana . 2007. “Improvement of Phylogenies After Removing Divergent and Ambiguously Aligned Blocks From Protein Sequence Alignments.” Systematic Biology 56: 564–577. 10.1080/10635150701472164. 17654362

[ede70061-bib-0024] Vlašánková, A. , E. Padyšáková , M. Bartoš , X. Mengual , P. Janečková , and Š. Janeček . 2017. “The Nectar Spur Is Not Only a Simple Specialization for Long‐Proboscid Pollinators.” New Phytologist 215: 1574–1581. 10.1111/nph.14677. 28677219

[ede70061-bib-0025] Wessinger, C. A. , and L. C. Hileman . 2020. “Parallelism in Flower Evolution and Development.” Annual Review of Ecology, Evolution, and Systematics 51: 387–408. 10.1146/annurev-ecolsys-011720-124511.

[ede70061-bib-0026] Whittall, J. B. , and S. A. Hodges . 2007. “Pollinator Shifts Drive Increasingly Long Nectar Spurs in Columbine Flowers.” Nature 447: 706–709. 10.1038/nature05857. 17554306

[ede70061-bib-0027] Wickham, H. 2016. ggplot2: Elegant Graphics for Data Analysis. Springer‐Verlag.

[ede70061-bib-0028] Wilson‐Sánchez, D. , N. Bhatia , A. Runions , and M. Tsiantis . 2022. “From Genes to Shape in Leaf Development and Evolution.” Current Biology 32: R1215–R1222. 10.1016/j.cub.2022.09.021. 36347226

[ede70061-bib-0029] Yaffe, H. , K. Buxdorf , I. Shapira , et al. 2012. “Logspin: A Simple, Economical and Fast Method for RNA Isolation From Infected or Healthy Plants and Other Eukaryotic Tissues.” BMC Research Notes 5: 45. 10.1186/1756-0500-5-45. 22260178 PMC3282632

[ede70061-bib-0030] Yang, M.‐W. , and X.‐F. Jin . 2023. “Diversity and Evolutionary Ecology of Nectar Spurs in Angiosperms.” Chinese Journal of Plant Ecology 47: 1193–1210. 10.17521/cjpe.2022.0445.

[ede70061-bib-0031] Yant, L. , S. Collani , J. Puzey , C. Levy , and E. M. Kramer . 2015. “Molecular Basis for Three‐Dimensional Elaboration of the *Aquilegia* Petal Spur.” Proceedings. Biological sciences 282: 20142778. 10.1098/rspb.2014.2778. 25673682 PMC4345449

