# Supplementary material for: Floral KNOX Expression Supports Parallel Evolution of Nectar Spurs in Snapdragon Relatives (Antirrhineae: Plantaginaceae)
Source: Evol Dev. 2026 Sep 14;28(3):e70061. doi: 10.1111/ede.70061 (PMC13572962; doi:10.1111/ede.70061)
Supplement: Supplementary file 1 [file EDE-28-e70061-s001.pdf]

Figure S1. Protein alignment of *HIRZ* and *INA* orthologues, with representative *STM*-like sequences.

```
                                 1
KNOX_Jovellana_KY284629.1        ---------- ---------- ---------- ---------- ----------
INA_Kickxia                      ---------- ---------- ---------- ---------- ----------
STM1_Loxostigma_KY284638.1       ---------- ---------- ---------- ---------- ----------
STM1_Streptocarpus_AY655754.1    MEGSAGNLNL NSYRGANSFM GFG---DNV- ---NG-FSP- ------MMMM
INA_Antirrhinum_AY072735.1       MEGGGGSSS- -----NSCMI GFGENTSSN- ---NG-FCP- ------MTMM
INA_Chaenorhinum                 ---------- ---------- ---------- ---------- ----------
INA_Linaria_JN005931.1           ---------- ---------- ----MSSNN- ---NG-LCP- ------MMMM
INA_Cymbalaria                   ---------- ---------- ---------- ---------- ----------
STM1_Tropaeolum_MW391954.1       MEVSS--NSK SSSTSVPCMI AFGDNNNNRS SSSNGVMYP- ------MMMM
HIRZ_Cymbalaria                  ---------- ---------- ---------- ---------- ----------
HIRZ_Kickxia                     ---------- ---------- ---------- ---------- ----------
HIRZ_Antirrhinum_AY072736.1      MEGSGGG--- -------GMM AFGENNNNN- --SNNGFCPS N-----MMMP
HIRZ_Linaria_JN005930.1          MEGGNSS--- -------SSL MAAFDDHRI- --NNFGFFPN SNISTSTMMM
STM_Arabidopsis_U32344.1         ME-------- SGSNSTSCPM AFAGDN---- --SDGPMCP- ------MMMM
HIRZ_Chaenorhinum                MEGVS----- -GTSNSLCVM AFGENNNNS- ---NG-FCP- ------STMM

                                51
KNOX_Jovellana_KY284629.1        ---------- ---------- ---------- ---------- ----------
INA_Kickxia                      ---------- --HPP----- --NGEAGNNN NNHNNTLFLP -LPSNDLNQQ
STM1_Loxostigma_KY284638.1       ---------- ---------- ---------- ---------- ----------
STM1_Streptocarpus_AY655754.1    MHANDP---- ---------- ---------N GDCSQPIFLP -LPAANHQGI
INA_Antirrhinum_AY072735.1       MMPPPSMASS ---------- ---------- HPNGNTLFFP -LPSTNNSHQ
INA_Chaenorhinum                 ---------- ---------- ---------- ---------- ----------
INA_Linaria_JN005931.1           MMPPQSTNNN NNNNN----- --NATGNNNN NNNNNTLYFP -LPSSTTTNR
INA_Cymbalaria                   ---------- ---------- ---------- ------LFXP -LPISTTXHS
STM1_Tropaeolum_MW391954.1       PLMSSHHQHE -Q--H----- --H------- GDASSSDYYS -IRTRNI---
HIRZ_Cymbalaria                  ---------- ---------- ---------- ---------- ----------
HIRZ_Kickxia                     ---------- ---------- ---------- ---------- ----------
HIRZ_Antirrhinum_AY072736.1      QLASNCE--- ---------- ---------- GGYSTTPFVP LVPFTNHHHH
HIRZ_Linaria_JN005930.1          MMPSCSQ--- ---------- ---------- SPNDTTSFLP PIPFTNHHHH
STM_Arabidopsis_U32344.1         MPPIMTSHQH HGHDHQHQQQ EHDGYAYQSH HQQSSSLFLQ -SLAPPQGTK
HIRZ_Chaenorhinum                MMPQMANEQV ---------- ---------- GYNNNTPFFS -LPFANHHQQ

                               101
KNOX_Jovellana_KY284629.1        ---------- ---------- ---------- -----STGYY FMESNN----
INA_Kickxia                      ---------- ---------- -NN------N SNTITSTGYY FMDGNE----
STM1_Loxostigma_KY284638.1       ---------- ---------- ---------- -------GYY FMESDG----
STM1_Streptocarpus_AY655754.1    NR-------N TGGGGGLTP- ---------E HHSNMSTGYY FMEGDG----
INA_Antirrhinum_AY072735.1       GL-------N HRTSASTMLE DQH------N NNTITSTGYY FMDGND----
INA_Chaenorhinum                 ---------- ---------- ---------- ---------- -----N----
INA_Linaria_JN005931.1           ---------- HSGTTSMMLE DQH------N --NITSTGYY FMENNG----
INA_Cymbalaria                   SS-------V GGGNSSMMFE DHXXNXXGGG NTTITSXGYY FMDGNN----
STM1_Tropaeolum_MW391954.1       ---------- --------PL LHHSTTPHQD HHLNTKNNTY FMDSNN----
HIRZ_Cymbalaria                  ---------- ---------- ---------- ---------- -----N----
HIRZ_Kickxia                     --------AP LVGGSSMML- ---------N NNSVNTTGYY FMD-------
HIRZ_Antirrhinum_AY072736.1      HQEMNRQRST VVGGSSMVYE EGH----QNN SNAITSTGYY FMDSTN----
HIRZ_Linaria_JN005930.1          QSDPINNKSP MIT----YQE ENH----HNN NNTITSTGYY FMNS-N----
STM_Arabidopsis_U32344.1         NK-------V ASS------- ---------S SPSSCAPAYS LMEIHHNEIV
HIRZ_Chaenorhinum                QQ-------- ---------- ----ETNQNN NNTITSTGYY FMDSDN----

                               151
KNOX_Jovellana_KY284629.1        ---DA--G-- --SCAVKAKI MAHPHYPRLL AAYVNCQKIG APPEVVARLE
INA_Kickxia                      ---DA--AAN NMNCSIKAKI MAHPHYHRLL AAYVNCQKIG APPDVVARLE
STM1_Loxostigma_KY284638.1       ---DA--G-- --SCSVKSKI MAHPHYPRLL AAYVNRQKIG APPEVVAKLE
STM1_Streptocarpus_AY655754.1    ---DA--G-- --GSSVKSKI IAHPHYPRLL AAYVSCQKIG APPEVVAKLE
INA_Antirrhinum_AY072735.1       ---------- --NDSVKAKI MAHPYYHKLL AAYINCQKIG APPEVAVKLE
INA_Chaenorhinum                 ---------- --NEDVKAKI MAHPHYHRLL AAYINCQKIG APPEVAARLE
INA_Linaria_JN005931.1           ---S------ NMDDSLKAKI MAHPHYHKLL ASFVNCQKIG APAEVTARLE
INA_Cymbalaria                   ---NN----I NIDDXVKAKI IAHPHYHRLL AAYVNCQKIG APPDVVARLE
STM1_Tropaeolum_MW391954.1       ---GSSSSAT EVSPVIKAKI MAHPHYNRLL AAYVNCQKVG APPEVIVRLE
HIRZ_Cymbalaria                  ---------- DSECSLKAKI MAHPHYHRLL AAYVNCHKIG APGEVVARLE
HIRZ_Kickxia                     ---------- --DCSLKAKI MAHPHYNRLL AAYVNCQKIG APAEVVARLE
HIRZ_Antirrhinum_AY072736.1      ---------- CDESSLKAKI MAHPHYHRLL AAYVNCHKIG APPEVVSRLE
HIRZ_Linaria_JN005930.1          ---------- ADDCSLKAKI MSHPHYHRLL AAYVNCQKIG APDEVVERLE
STM_Arabidopsis_U32344.1         AGGINPCSSF SSSASVKAKI MAHPHYHRLL AAYVNCQKVG APPEVVARLE
HIRZ_Chaenorhinum                ---------- --DCSLKAKI MAHPHYQRLL TAYVNCQKIG APPEVVARLE
```

KNOX

```
201
KNOX_Jovellana_KY284629.1          EAYASATGM- ---CRSGTSC VGEDPALDQF MEAYCEMLTK YEQELSKPFK
INA_Kickxia                        ASAAAAAI-- ---SRHGTIS VGEDPALDQF MEAYCEMLSK YEQELSKPFR
STM1_Loxostigma_KY284638.1         EACASATAI- ---SRNDRSF VGEDPALDQF MEAYCEMLTK YEQELSKPFK
STM1_Streptocarpus_AY655754.1      EVCASATSTG ---CRNERSC VGEDPALDQF MEAYCGMLTK YEQELSKPFK
INA_Antirrhinum_AY072735.1         EACASAATMG ---RN-SVSR IGEDPALDQF MEAYCEMLSK YEQELSKPFR
INA_Chaenorhinum                   EACASAAVIG ---RN-NTSC IGEDPSLDQF MEAYCDMLTK YEQELSKPFK
INA_Linaria_JN005931.1             EACSSAASIG ---RGHGTSC IGEDPALDQF MEAYCEMLTK YEQGLSKPFR
INA_Cymbalaria                     EACGSAAATM ---G-GRNSR IGEDPGLDQF MEAYCEMLTK YEQELSKPFR
STM1_Tropaeolum_MW391954.1         EACSSAAMVN NGGSSSTTSC LGEDPALDQF MEAYCEMLIK YEQELSKPFK
HIRZ_Cymbalaria                    ELSTAASAMV R--RGGTTTS IGEDPALDQF MEAYSEMLTK YEQELSKPFK
HIRZ_Kickxia                       EASSSAAASV G--RH-GTVC LGEDPALDQF MEAYSEMLTK YEQELSKPFK
HIRZ_Antirrhinum_AY072736.1        EAAA---AMA R--H--GTIS VGEDPGLDQL MEAYSEMLSK YEQELSKPFK
HIRZ_Linaria_JN005930.1            EAAAMCRS-- H--GLQGSIC VGEDPSLDQF MEAYSEMLTK YEQELSKPLK
STM_Arabidopsis_U32344.1           EACSSAAAAA A--SMGPTGC LGEDPGLDQF MEAYCEMLVK YEQELSKPFK
HIRZ_Chaenorhinum                  VAASSM---- ---GRNGTIR LGDDPALDQF MEAYAEMLTK YEQELSKPFK
```

```
251
KNOX_Jovellana_KY284629.1          EAMIFLSRIE CQFQALTLDP S-DSGACGGE AIDR--IGSS EEDVDINN-S
INA_Kickxia                        EAMLFLSRIE CQFKALTLSS S--DSACGEA -MDR--NGSS EEEVDVNN-S
STM1_Loxostigma_KY284638.1         EAMLFLSRIE CQFKALNLSH S-DSGACGEA -VDR--NGSS EEEFDVNN-S
STM1_Streptocarpus_AY655754.1      DAMLFFSRFE CQFKALTLSH SADSGACDEA VLEQ--NGSS EEEFDVNN-S
INA_Antirrhinum_AY072735.1         EAMLFLSRIE CQFKALTLSS S--DSACGEA -MDR--HGSS EEEIDVDN-S
INA_Chaenorhinum                   EAMLFLSRME CQFKTLTVSS S--DSACGDA -MDR--QGSS EEEVDVNN-S
INA_Linaria_JN005931.1             EAMLFLSRME CQFKALTVSS S-DNSACGDA -MDR--QGSS EEEADMNN-N
INA_Cymbalaria                     EAMLFLTRIE SQFKAITLSS S--QSACGEA -MDR--HGSS EEXVDVNN-S
STM1_Tropaeolum_MW391954.1         EAMLFLQRVE SQFKALSFSP S-STSACVEA TDHR--SASS EEELDVNN-D
HIRZ_Cymbalaria                    EALLFLSTID SQFKALTLSS H----SARGE AMYG--NGSS EEEIDMNN-S
HIRZ_Kickxia                       EALHFLTTFE SHFKALTLSP SSHSGPARGE AMYR--NGSS EEEMDVNNMS
HIRZ_Antirrhinum_AY072736.1        EAMLFLSRIE SQFKALTVS- -----AARGE AMFR--NGSS EEEIDVNN-S
HIRZ_Linaria_JN005930.1            EAMLFFSRIE SQFKAIALSH PSPAATARGE ALYR--NGSS EEEIDVNN-S
STM_Arabidopsis_U32344.1           EAMVFLQRVE CQFKSLSLSS PSSFSGYGET AIDRNNNGSS EEEVDMNN-E
HIRZ_Chaenorhinum                  EAMLFLSRFE SQFKALTLSP SD-HSARGGE ALHR--NGSS EEEIDVNN-S
```

ELK

```
301
KNOX_Jovellana_KY284629.1          FIDPQAEDRE LKGQLLKKYS GYLGSLKQEF MKKRKKGKLP KEARQQLLEW
INA_Kickxia                        FFDPQAEDRE LKGQLLRKYS GYLGSLKQEF MKKRKKGKLR KEARQQLLDW
STM1_Loxostigma_KY284638.1         FIDPQAEDRE LKGQLSRKYS GYLGNLKQEF MKKRKKGKLP KEARQQLLDW
STM1_Streptocarpus_AY655754.1      FIDPQAEDHE LKGQLLRKYS GYLGNLKQEF MKKRKKGKLP KEARQQLLDW
INA_Antirrhinum_AY072735.1         LIDPQAEDRE LKGQLLRKYS GYLGSLKQEF MKKRKKGKLP KEARQQLLDW
INA_Chaenorhinum                   FIDPQAEDRE LKGQLLRKYS GYLGSLKQEF MKKRKKGKLP KEARQQLLDW
INA_Linaria_JN005931.1             FIDPQAEDRE LKGQLLRKYS GYLGSLKQEF MKKRKKGKLP KEARQQLLDW
INA_Cymbalaria                     FIDPQAEDQE LKGQLLRKYS GYLGNLKQEF MKKRKKGKLP KEARQQLLEW
STM1_Tropaeolum_MW391954.1         TIDPQAEDRE LKGQLLRKYS GYLGSLKQEF MKKRKKGKLP KEARQQLLDW
HIRZ_Cymbalaria                    FIDPQAEDVE LKGQLLRKYS GYLGSLKQEF MKKRKKGKLP KEARQQLLEW
HIRZ_Kickxia                       LIDPQAEDIE LKGQLLRKYS GYLGSLKQEF MKKRKKGKLP KEARQQLLEW
HIRZ_Antirrhinum_AY072736.1        FIDPQAEDIE LKGQLLRKYS GYLGSLKQEF MKKRKKGKLP KEARQQLLEW
HIRZ_Linaria_JN005930.1            MIDPQAEDVE LKGQLFRKYS GYLGSLKQEF MKKRKKGKLP KEARQQLLDW
STM_Arabidopsis_U32344.1           FVDPQAEDRE LKGQLLRKYS GYLGSLKQEF MKKRKKGKLP KEARQQLLDW
HIRZ_Chaenorhinum                  FIDPQAEDVE LKGQLLRKYS GYLGSLKQEF MKKRKKGKLP KEARQQLLEW
```

Homeodomain

```
351
KNOX_Jovellana_KY284629.1          WSRHYNCPYP SESQKLALAE STGLDQKQIN NWFINQRKRH WKPSEDMQFV
INA_Kickxia                        WSRHYKWPYP SESQKLALAE STGLDQKQIN NWFINQRKRH WKPSEDMQFV
STM1_Loxostigma_KY284638.1         RSRHYKWPYP SESQKLALAE STGLDQKQIN NWFINQRKRH WKPSEDMQFV
STM1_Streptocarpus_AY655754.1      WSRHYKWPYP SESQKLALAE STGLEQKQIN NWFINQRKRH WKPSEDMQFV
INA_Antirrhinum_AY072735.1         WSRHYKWPYP SESQKLALAE QTGLDQKQIN NWFINQRKRH WKPSEDMQFV
INA_Chaenorhinum                   WSRHYKWPYP SESQKLMLAE STGLDQKQIN NWFINQRKRH WKPSEDMQFV
INA_Linaria_JN005931.1             WSRHYKWPYP SESQKLALAE STGLDQKQIN NWFINQRKRH WKPSEDMQFV
INA_Cymbalaria                     WSRHYKWPYP SESQKLALAE STGLDQKQIN NWFINQRKRH WKPSEDMQFV
STM1_Tropaeolum_MW391954.1         WSRHYKWPYP SEQQKLALAE STGLDQKQIN NWFINQRKRH WKPSEDMQFV
HIRZ_Cymbalaria                    WSRHYKWPYP SESQKLALAE STGLDQKQIN NWFINQRKRH WKPSEDMQFV
HIRZ_Kickxia                       WSRHYKWPYP SESQKLALAE STGLDQKQID NWFINQRRRH WKPSEDMQFV
HIRZ_Antirrhinum_AY072736.1        WSRHYKWPYP SESQKLALAE STGLDQKQIN NWFINQRKRH WKPSEDMQFV
HIRZ_Linaria_JN005930.1            WSRHYKWPYP SESQKLALAE STGLDQKQIN NWFINQRKRH WKPSEDMQFV
STM_Arabidopsis_U32344.1           WSRHYKWPYP SEQQKLALAE STGLDQKQIN NWFINQRKRH WKPSEDMQFV
HIRZ_Chaenorhinum                  WSRHYKWPYP SESQKLALAE STGLDQKQIN NWFITKK--- ----------
```

```
                                   401
KNOX_Jovellana_KY284629.1          VM-------- ---------- ---------- ----
INA_Kickxia                        VMDAAH--PG HYFMDN--VL GNPFSMDISP ALL-
STM1_Loxostigma_KY284638.1         VMDAAH--P- HYYMDN--IM GSPFPMDIS- ----
STM1_Streptocarpus_AY655754.1      VMDAAH--P- HYYMDN--IM ATPFPMDISS SFL-
INA_Antirrhinum_AY072735.1         VMDAAH--PG HYYMDN--VL GNPFAMDILP TLL-
INA_Chaenorhinum                   VMDAAH--PG HYYMDS--VL GNPFAMDISP ALL-
INA_Linaria_JN005931.1             VMDAAH--PG HYYMDN--AL GNPFAMDISP SFL-
INA_Cymbalaria                     VMDAAH--PG HYYMDN--VL GNPFAMDISP QLL-
STM1_Tropaeolum_MW391954.1         VMDATH--S- HYYMDN--VM GNPFPMDISP SLL-
HIRZ_Cymbalaria                    VMDATN---P QYYMENHHLL GNPFPMDISP PALL
HIRZ_Kickxia                       VMDAAN---P QYYMEN--VM GNPFPMDISP AFL-
HIRZ_Antirrhinum_AY072736.1        VMDAAN---P QYYMEN--IL GNPFPMDISP ALL-
HIRZ_Linaria_JN005930.1            VMDAAAANPQ QYSYMDHNVL GNPFPMDISP ALL-
STM_Arabidopsis_U32344.1           VMDATH--PH HYFMDN--VL DNPFPMDHIS STML
HIRZ_Chaenorhinum                  ---------- ---------- ---------- ----
```
