# Supplementary material for: Floral KNOX Expression Supports Parallel Evolution of Nectar Spurs in Snapdragon Relatives (Antirrhineae: Plantaginaceae)
Source: Evol Dev. 2026 Sep 14;28(3):e70061. doi: 10.1111/ede.70061 (PMC13572962; doi:10.1111/ede.70061)
Supplement: Supplementary file 2 [file EDE-28-e70061-s002.pdf]

Table S1. Gene sequences used for phylogenetic inference and orthology assessment of *HIRZ* / *INA* sequences.

Organism	Gene	Accession	Repository
Antirrhinum majus	HIRZ	AY072736.1	NCBI
Antirrhinum majus	INA	AY072735.1	NCBI
Linaria vulgaris	HIRZ	JN005930.1	NCBI
Linaria vulgaris	INA	JN005931.1	NCBI
Streptocarpus saxorum	STM1	AY655754.1	NCBI
Loxostigma griffithii	STM1	KY284638.1	NCBI
Jovellana punctata	KNOX	KY284629.1	NCBI
Tropaeolum longifolium	STM1	MW391954.1	NCBI
Arabidopsis thaliana	STM	U32344.1	NCBI
Misopates orontium	HIRZ	ENSMORG00010006892.1	Ensembl (DTOL)
Misopates orontium	INA	ENSMORG00010003330.1	Ensembl (DTOL)

Table S2. Primers used for nrITS checks, gene-specific RT-PCR and qRT-PCR analyses.

Primer name	Gene /Region	Sequence (5' to 3')	Tm (°C)	Product size (bp)
ITSleu1*	ITS	GTCCACTGAACCTTATCATTTAG	55	
ITS4*	ITS	TCCTCCGCTTATTGATATGC	56	~750
HIRZ_RT_F5	HIRZ	TACAGTGGGTATTTGGGCAGC	60.34	
HIRZ_RT_R1	HIRZ	CAGTGCCGTTTCCTTTGGTT	59.26	218
INA_RT_F5	INA	ATGGAGGCTTATTGTGAAATGC	61.6	
INA_RT_R5	INA	TCTTCATGAATTCCTGCTTGAGG	61.12	292
Kick_TUA5_F1	TUA5	CCATTTACCCTTCCCCTCAGG	59.78	
Kick_TUA5_R1	TUA5	ACGGAGAGAAGCGGTCAAAG	60.04	221
Cymb_TUA5_F1	TUA5	CCATCTACCCTTCCCCACAGG	59.3	
Cymb_TUA5_R1	TUA5	ATCAACATTGAGGGCACCGT	57.1	245
Chaen_TUA5_F3	TUA5	CGCAAGTTTCGACTGCTGTT	59.7	
Chaen_TUA5_R3	TUA5	CCCCATCAAAGCGGAGTGAA	60.32	216

*Universal primers taken from Bohs, L. (2007). Phylogeny of the Cyphomandra clade of the genus *Solanum* (Solanaceae) based on ITS sequence data. *Taxon* 56:1012-1026.
